# C/EBPβ transcription factor promotes alcohol-induced liver fibrosis in males via HDL remodeling

**DOI:** 10.1097/HC9.0000000000000645

**Published:** 2025-02-19

**Authors:** Michael Schonfeld, Kruti Nataraj, Steven Weinman, Irina Tikhanovich

**Affiliations:** 1 Department of Internal Medicine, University of Kansas Medical Center, Kansas City, Kansas, USA; 2 Kansas City VA Medical Center, Kansas City, Missouri, USA

**Keywords:** ALD, apolipoprotein, epigenetic changes, inflammation, macrophages

## Abstract

**Background:**

Alcohol-associated liver disease (ALD) is the main cause of alcohol-associated mortality. However, the mechanism of ALD development is poorly understood. Epigenetic changes are thought to play an important role in ALD. We aimed to define the epigenetic changes induced by alcohol and predict drivers of these changes.

**Methods:**

Mice were fed high-fat diet with or without 20% of alcohol in the drinking water for 20 weeks (WDA model). scATAC-seq data set was analyzed using Signac R package. To test the role of C/EBPβ, Cebpb-floxed mice were treated with AAV8-TBG-Cre or AAV8-control.

**Results:**

We analyzed differentially accessible regions in livers from control and alcohol-fed mice and found that activity of C/EBPβ transcription factor was associated with alcohol-induced epigenetic changes in hepatocytes. C/EBPβ protein levels were significantly upregulated in multiple models of ALD and human ALD samples. Using hepatocyte-specific Cebpb knockout mice we found that Cebpb loss protected male mice from alcohol-induced fibrosis development. We found no protection in female mice, suggesting that this mechanism is specific to male ALD. In vitro studies suggested that the protective effect of Cebpb loss was mediated by altered hepatocyte—macrophage cross talk. Cebpb knockout in hepatocytes reduced a profibrotic and promoted a pro-resolving phenotype in macrophages, thus modulating ALD development. We further identified the mediators of the cross talk. Cebpb knockout altered the expression of several HDL protein components, increasing APOA1 and apolipoprotein M and reducing apolipoprotein E and SAA levels in male mice. HDL secreted by Cebpb knockout hepatocytes was sufficient to confer anti-inflammatory and antifibrotic changes to macrophages.

**Conclusions:**

Taken together, alcohol-induced C/EBPβ activation is a key driver of ALD fibrosis in males via C/EBPβ-dependent HDL remodeling.

## Graphical abstract

**Figure:**
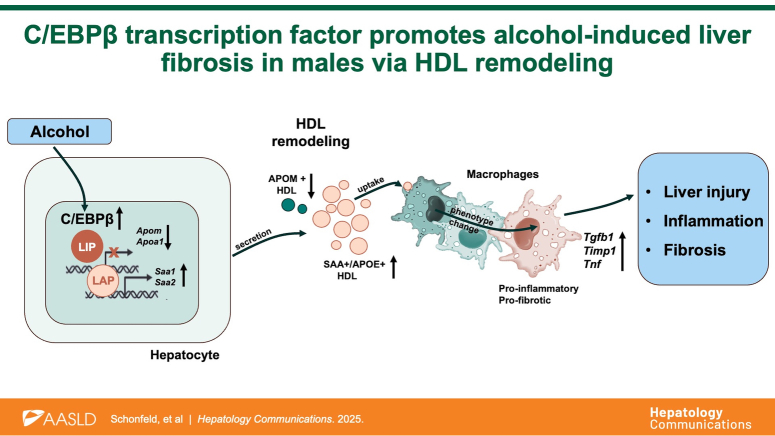

## INTRODUCTION

Alcohol-associated liver disease (ALD) encompasses a spectrum of disorders that commonly progress from steatosis to steatohepatitis with fibrosis and ultimately to cirrhosis. Cirrhosis is the ninth leading cause of death in the United States and about 35%–50% of cirrhosis deaths are alcohol associated.^1^ In ALD, multiple liver cell types are impacted by alcohol exposure; however, it is not clear if the effects of alcohol on individual cells are direct, or a response to alcohol-induced signals from other cells. Advances in technologies such as single-cell sequencing and spatial transcriptomics have revealed new details regarding the complexity of hepatic cell types^2^ and have highlighted the importance of cell-cell communication via secreted mediators such as peptides, lipids, hormones, and cytokines in disease development and progression.^3^ Recent studies suggest that hepatocytes play a central role in driving ALD and influence the phenotype of nonparenchymal cells in the liver both during disease development^4^ and resolution.^5^

Recently, we identified the H3K4-specific demethylases KDM5B and KDM5C as mediators of alcohol-induced epigenetic changes that promote ALD development. However, KDM5 demethylase chromatin binding requires recruitment by specific transcription factors (TF), since demethylases themselves do not have DNA binding specificity. In this work, we identified the C/EBPβ TF as a potential effector of alcohol-induced epigenetic changes.

C/EBPβ is a liver-enriched TF. Its protein level is low/undetectable under control conditions, but it gets rapidly induced after liver injury as part of a regeneration program.^6^,^7^,^8^,^9^,^10^,^11^ It promotes acute response genes and suppresses metabolic genes, transcriptionally antagonizing HNF4α activity.^6^,^12^ C/EBPβ is activated by a variety of signals that play a role in the regulation of multiple inflammatory pathways.^6^,^7^,^8^,^13^,^14^ It can be activated by IL-17A, IL-10, IL-27, HGF, and IGFBP-1, as well as other factors including caloric restriction, beta-adrenergic receptor signaling, and cAMP.^8^,^14^,^15^ C/EBPβ is a multifunctional TF that controls metabolism, inflammation, and cell cycle progression.^6^,^7^,^8^,^9^,^10^,^11^ It has an important role in orchestrating a regeneration program in the liver after liver injury. It does so by timely altering target expression utilizing multiple protein isoforms (liver-enriched activating protein [LAP]) and liver-enriched inhibitory protein [LIP] and post-translational modifications.^10^,^13^,^15^,^16^,^17^ Previous studies have reported that C/EBPβ dysregulation is involved in cancer,^13^,^17^ lifespan shortening,^6^ and acute-on-chronic liver failure.^12^

Here we report that C/EBPβ is induced by alcohol and that hepatocyte C/EBPβ activity is required for alcohol-induced fibrosis development in male mice. We further identified hepatocyte-to-macrophage communication as an important driver of C/EBPβ-dependent fibrosis development and HDL as the key component of cell-cell communication between hepatocytes and macrophages during ALD development.

## METHODS

### Mice

*Cebpb-*floxed mice (BALB/cJ-Cebpb^tm1.1Elgaz^) were obtained from Jackson lab and backcrossed for 7 generations to the C57BL6/J background. All mice were housed in a temperature-controlled, specific pathogen-free environment with 12-hour light-dark cycles. All animal handling procedures were approved by the Institutional Animal Care and Use Committee at the University of Kansas Medical Center (Kansas City, KS).

For fibrosis induction mice were treated with 200 mg/L of thioacetamide (TAA) in the drinking water for 2 months. Recombinant Angiopoietin-1 or saline control was injected i.p. at 3 µg/mouse.

### Western diet with alcohol model

For the previously described western diet alcohol model,^18^ both male and female mice were fed ad libitum western diet (Research Diets Inc., Cat# D12079B), and alcohol was given ad libitum in water. Mice received progressively increasing amounts of alcohol in water (3%, 10%, 15%, and 20% for 3 d each). After reaching 20%, mice continued for 12–18 weeks as indicated. Alcohol-containing water was changed twice weekly.

### Vectors

AAV-TBG-control and AAV-TBG-iCre were from VectorBiolabs, Malvern, PA, and were used at 1×10^11^ genome copies per mouse (Cre/control).

### Antibodies

Anti-COL1A1 antibodies were from Cell Signaling (COL1A1 (E8I9Z) Rabbit mAb #91144). Anti-C/EBPβ (C/EBP beta Antibody (H-7): sc-7962), anti-APOM, and anti-apolipoprotein E (APOE) antibodies were from SantaCruz. Anti-SAA (serum amyloid A) monoclonal antibody was from Novus.

### Analysis of blood samples

Whole blood collected from the retroorbital vein of mice was used to measure prothrombin time (CoaguChek XS system, 04625315160, Roche). Serum was used to measure ALT (Pointe Scientific ALT Liquid Reagents, A7526150, Pointe Scientific) and AST (Pointe Scientific ALT Liquid Reagents, A7561450, Pointe Scientific).

### RNA-Seq

For RNA-Seq analysis, total RNA was isolated from the liver using the Qiagen RNA isolation kit. Three individual mice were used per condition. Library generation and sequencing were performed by BGI genomics services (BGI, Cambridge, MA). Twenty-seven samples were sequenced using the BGISEQ platform, generating an average of ~4.57G Gb bases per sample. Hierarchical indexing for spliced alignment of transcripts was used to align the clean reads to the reference genome. Bowtie2 was used to align the clean reads to the reference genes. The average mapping ratio with a reference genome (GRCm38.p6) was 96.14%, and 16,869 genes were identified. Differential gene expression was identified with DESeq. 2. RNA-seq raw data can be found under GSE276692.

### Human samples

Deidentified human specimens were obtained from the Liver Center Tissue Bank at the University of Kansas Medical Center. All studies using human tissue samples were approved by the Human Subjects Committee of the University of Kansas Medical Center.

### Cell isolation

Mouse livers were digested by retrograde perfusion with Liberase TM via the inferior vena cava. The dissociated cell mixture was placed into a 50 mL conical tube and centrifuged twice at 50*g* for 2 minutes to pellet hepatocytes. The nonparenchymal cell-containing cell supernatant was further used to isolate kupffer cells (KC), LSEC, and HSC. The cell suspension was pelleted by centrifugation (700*g*, 10 min, 4 °C) and resuspended in PBS and OptiPrep (Sigma) to a final concentration of 17%. Afterward, 5 mL of the indicated suspension was placed in a 15 mL polystyrene conical centrifuge tube (BD Biosciences) and overlaid with 5 mL of a 9% OptiPrep solution followed by 2 mL PBS. After centrifugation at 1400*g* for 20 minutes at 4 °C with decreased acceleration and without breaks, the various cell types were arranged according to their density. HSCs were enriched in the upper cell layer, whereas KC and LSEC were separated as a second layer of higher density. Cell fractions were collected separately by pipetting. HSC purity over 99% was confirmed by retinoid-based FACS sorting. The KC/LSEC fraction was pelleted, KCs and endothelial cells were isolated with F4/80+ and CD146+MicroBeads (MiltenyiBiotec), respectively, according to the manufacturer’s instructions. Cells were applied onto LS magnetic-activated cell sorting columns (MiltenyiBiotec), which were placed within the magnetic field of a magnetic-activated cell sorting separator and washed 3 times with magnetic-activated cell sorting buffer (MiltenyiBiotec). Cells were eluted and were then seeded into culture dishes. Endothelial cells were seeded on dishes coated with collagen-I.

### Trans-well co-culture

For co-culture experiments, primary LSECs were placed in cell inserts of 24 well trans-well (Corning Incorporated, Acton, MA, 0.4 µm pore size) at a seeding density of 5 × 10^4^/well. Cells were treated as indicated. Freshly isolated hepatocytes were seeded in the bottom well at a seeding density of 1 × 10^5^/well. The cells were then cultured for 24 hours, and hepatocytes were harvested for RNA isolation. Alternatively, primary hepatocytes from *Cebpb*-floxed mice were placed in cell inserts. Cells were transfected with Cre recombinase expressing vector, empty vector control, or HNF4α expressing plasmid. Freshly isolated liver macrophages were seeded in the bottom well. The cells were then cultured for 24 hours, and macrophages were harvested for RNA isolation. Alternatively, macrophages were used for a collagen degradation assay, using DQ Collagen (ThermoFisher, Cat# D12060), type I From Bovine Skin, and Fluorescein Conjugate, according to manufacturer’s instructions. Cells were incubated in 50 mM Tris-HCl (pH 7.6), 150 mM NaCl, 5 mM CaCl2, 0.5% agar, and 10 mg/L DQ Collagen for 2 hours at 37 °C. Digestion product fluorescence was measured at 525 nm.

### Immunohistochemistry

Liver tissue sections (5 μm thick) were prepared from formalin-fixed, paraffin-embedded samples. Immunostaining on formalin-fixed sections was performed by deparaffinization and rehydration, followed by antigen retrieval achieved by heating in a pressure cooker (121 °C) for 5 minutes in 10 mM sodium citrate, pH 6.0, as described.^19^ Peroxidase activity was blocked by incubation in 3% hydrogen peroxide for 10 minutes. Sections were rinsed three times in PBS/PBS-T (0.1% Tween-20) and incubated in Dako Protein Block (Dako) at room temperature for 1 hour. After the removal of the blocking solution, slides were placed into a humidified chamber and incubated overnight with a primary antibody diluted 1:300 in Dako Protein Block at 4 °C. The antigen was detected using the SignalStain Boost IHC detection reagent (catalog # 8114; Cell Signaling Technology, Beverly, MA), developed with diaminobenzidene (Dako, Carpinteria, CA), counterstained with hematoxylin (Sigma-Aldrich), and mounted.

### RT-PCR

RNA was extracted from livers using the RNeasy Mini Kit (Qiagen). Complementary DNA was generated using the RNA reverse transcription kit (Applied Biosystems, Cat.No 4368814). Quantitative real-time reverse transcription followed by PCR was performed in a CFX96 real-time system (Bio-Rad) using specific sense and antisense primers combined with iQ SYBR Green Supermix (Bio-Rad) for 40 amplification cycles: 5 seconds at 95 °C, 10 seconds at 57 °C, 30 seconds at 72 °C. mRNA concentrations were calculated relative to *Actb*.

### Primers

**Table:** 

mActb fwd	ATGTCACGCACGATTTCCCT
mActb rvs	CGGGACCTGACAGACTACCT
mTnf fwd	CTGAGACATAGGCACCGCC
mTnf rvs	CAGAAAGCATGATCCGCGAC
mCol1a1 fwd	TGGCCAAGAAGACATCCCTG
mCol1a1 rvs	GGGTTTCCACGTCTCACCAT
mCebpb fwd	TCACTTAAAGATGTTCCTGCGG
mCebpb rvs	TGCTCGAAACGGAAAAGGTTC
mTimp1 fwd	GTAAGGCCTGTAGCTGTGCC
mTimp1 rvs	AGCCCTTATGACCAGGTCCG
mTgfb1 fwd	TACGTCAGACATTCGGGAAGC
mTgfb1 rvs	TTTAATCTCTGCAAGCGCAGC
mCcl2 fwd	ACCTGGATCGGAACCAAATGAG
mCcl2 rvs	GCTGAAGACCTTAGGGCAGAT
mMmp12 fwd	GTGGTACACTAGCCCATGCTT
mMmp12 rvs	TCCACGTTTCTGCCTCATCAA
mStab2 fwd	CCAGCTGGGTAAATGCAACA
mStab2 rvs	ATATGACGGCTGGTGTCCTC
mIcam1 fwd	TCACCGTGTATTCGTTTCCG
mIcam1 rvs	GGTGAGGTCCTTGCCTACTTG

### HDL-C measurement

HDL-Cholesterol levels in serum were measured using Mouse HDL-Cholesterol Assay Kit (Crystal Chem) according to the manufacturer’s instructions.

### HDL isolation

HDL was isolated using HDL Purification Kit (Cell Biolabs Inc.) according to the manufacturer’s instructions. HDL was dialyzed against PBS overnight and used at 20 µg/mL. HDL purity was confirmed by gel electrophoresis in denaturing and non-denaturing conditions.

### Western blotting

Protein extracts (50 µg) were subjected to 10% SDS-PAGE, electrophoretically transferred to nitrocellulose membranes (Amersham Hybond ECL, GE Healthcare), and blocked in 3% bovine serum albumin/PBS at room temperature for 1 hour. Primary antibodies were incubated overnight at manufacturer-recommended concentrations. Immunoblots were detected with the ECL Plus Western Blotting Detection System (Amersham Biosciences, Piscataway, NJ) with the ODYSSEY Fc, Dual-Mode Imaging system (Li-COR).

### Statistics

Data were plotted and analyzed in Prism GraphPad. Comparison between different data sets was made using unpaired two-tailed *t* test with Welch correction and one-way ANOVA with Tukey post hoc test. *p*<0.05 was considered to be statistically different.

### Data availability statement

Raw data for all reported data sets are available upon request.

## RESULTS

### C/EBPβ expression and activity are induced by alcohol

**FIGURE 1:**
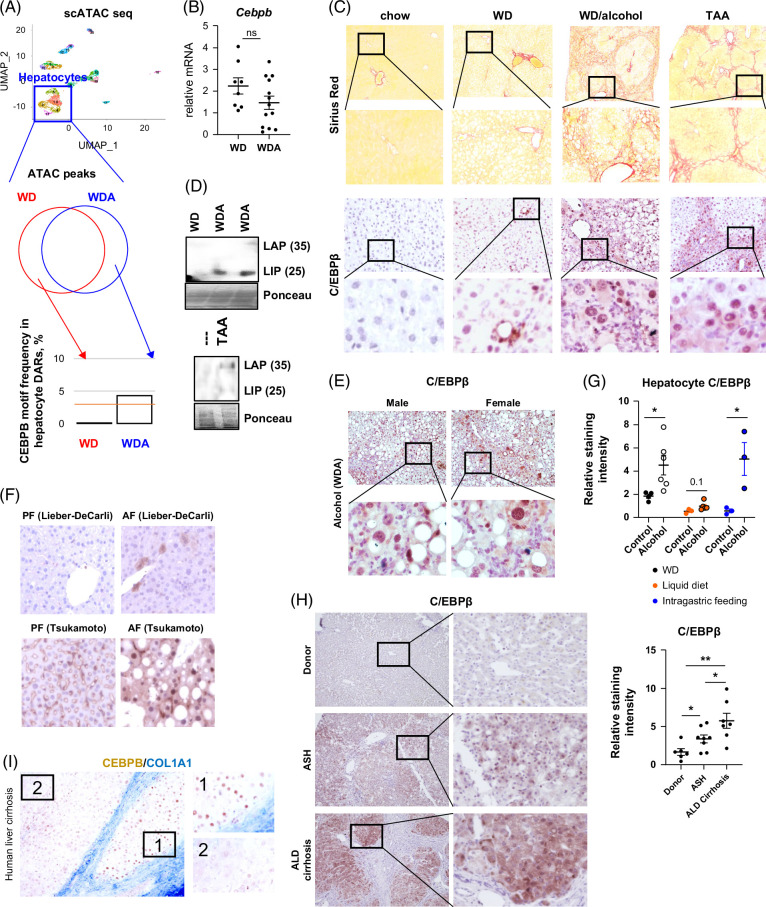
C/EBPβ is activated in models of liver disease. (A) scATAC-seq analysis (BioProject PRJNA1022784). Single-cell ATAC-seq analysis of liver cells from mice fed WD for 16 weeks (N=1) and WD with alcohol in the drinking water for 16 weeks (N=2, WDA). UMAP clustering of liver cells and motif enrichment analysis. C/EBPβ motif frequency in differentially accessible regions (DARs) from indicated comparison groups was analyzed using Transcription Factor Binding Site (TFBS) Analysis (https://github.com/ge11232002/TFBSTools). (B) Relative *Cebpb* mRNA in mice fed WD or WD with alcohol in the drinking water. (C) Mice were fed chow diet, WD, WDA with alcohol in the drinking water, or TAA in the drinking water to induce liver fibrosis. Sirius red staining and C/EBPβ staining in these mice. (D) Whole liver extracts from mice fed WD or WDA, water, or TAA were analyzed by western blot analysis using C/EBPβ specific antibodies. Bands with molecular weight around 25 kDa (LIP) and 35 kDa (LAP) were detected. (E) C/EBPβ staining in male and female mice fed WDA after 16 weeks. (F, Top) Representative images C/EBPβ staining in mice fed LieberDeCarli alcohol liquid diet for 8 weeks or pair-fed controls. Bottom, C/EBPβ staining in livers of mice fed alcohol using Tsukamoto intragastric feeding model or pair-fed controls.^22^ (G) Relative staining intensity of C/EBPβ in models of alcohol feeding. C/EBPβ nuclear staining in hepatocytes was measured using Aperio ImageScope. Hepatocyte nuclei were defined as large round nuclei with a diameter above 5 µm. N ≥3 per group. *, *p*<0.05. (H, I) Human liver cirrhosis samples were stained for C/EBPβ and COL1A1. Relative staining intensity of C/EBPβ nuclear staining in hepatocytes in human samples. N ≥5 per group. *, *p*<0.05, **, *p*<0.01. Abbreviations: ASH, alcohol-associated steatohepatitis; ATAC-Seq, assay for transposase-accessible chromatin with sequencing; C/EBPβ, CCAAT enhancer binding protein beta; LAP, liver-enriched activating protein; LIP, liver-enriched inhibitory protein; TAA, thioacetamide; WD, western diet; WDA, western diet with alcohol.

To analyze epigenetic changes induced by alcohol exposure, we used a recently performed single-cell assay for transposase-accessible chromatin with sequencing analysis.^20^ Mice were fed the high-fat western diet with alcohol in the drinking water (WDA) or plain water (WD) as a control, as described.^18^ In hepatocyte clusters we performed differential accessible regions analysis using Signac R package followed by TF motif enrichment analysis, as described.^21^ TF C/EBPβ motif was not present in WD control differential accessible regions, while the motif frequency was around 5% in differential accessible regions from alcohol-exposed hepatocytes (Figure 1A), suggesting that C/EBPβ is activated by alcohol. Interestingly, *Cebpb* gene accessibility and bulk mRNA levels (Figure 1B) were not significantly changed by alcohol.

We next examined C/EBPβ protein expression. Under normal, chow-fed conditions, protein expression of C/EBPβ was low. It was slightly elevated in WD-fed mice and further elevated in mice fed WD and alcohol (Figure 1C). The strongest staining occurred in the areas of liver fibrosis and inflammation. This was also apparent in a model of liver fibrosis induced by TAA where C/EBPβ protein level was elevated in hepatocytes adjacent to fibrotic septa (Figure 1C). We confirmed C/EBPβ upregulation by western blot analysis (Figure 1D). In WDA mice, we observed that the levels of C/EBPβ were similar between males and females (Figure 1E). In another model of ALD, C/EBPβ was mildly elevated in Lieber-DeCarli alcohol liquid diet-fed mice compared to pair-fed controls (Figure 1F). The weak positive staining was present in the pericentral zone, which correlates with mild liver injury in these mice. In contrast, Tsukamoto intragastric feeding model produces an alcohol-associated hepatitis-like phenotype and results in strong hepatocyte cytosolic and nuclear staining for C/EBPβ (Figure 1F, G). Interestingly, in pair-fed animals, C/EBPβ staining was also elevated, but exclusively in nonparenchymal cells, suggesting cell type–specific roles for C/EBPβ in liver disease. Finally, we found that in human alcohol-associated steatohepatitis and ALD-cirrhosis samples, C/EBPβ staining was significantly elevated compared to donor livers (Figure 1H). Moreover, in liver cirrhosis C/EBPβ nuclear staining was greater in hepatocytes adjacent to fibrotic septa and was lower in areas without fibrosis (Figure 1I).

Overall, we observed that C/EBPβ protein levels were elevated in liver disease models proportionally to the severity of liver disease and localized close to injury/fibrotic areas.

### C/EBPβ is protective in TAA-treated mice

**FIGURE 2:**
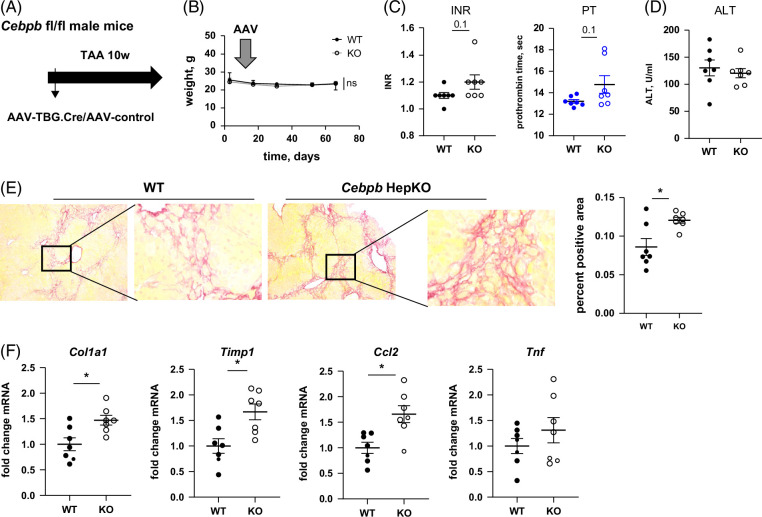
Hepatocyte-specific *Cebpb* knockout promotes TAA-induced fibrosis development. (A) Seven- to 8-week-old *Cebpb-*floxed male mice were treated with TAA for 10 weeks. After the start of feeding mice received 10^11^ gc/mouse of AAV-TBG-Cre or AAV-TBG-control. (B) Body weight in WT and KO mice. (C) PT and INR at the end of the experiment. (D) Serum ALT at the end of the experiment in WT and KO mice. (E) Representative images of Sirius Red staining and Sirius Red positive area (right). N ≥3 per group. *, *p*<0.05. (F) Relative mRNA in mice. N ≥3 per group. *, *p*<0.05. Abbreviations: AAV, adeno-associated virus; C/EBPβ, CCAAT enhancer binding protein beta; INR, international normalized ratio; KO, knockout; PT, prothrombin time; TAA, thioacetamide; WT, wild type.

Since C/EBPβ protein levels were elevated in multiple models of liver disease (Figure 1), we first tested the effect of *Cebpb* knockout (KO) in TAA toxicity, a liver disease model that is mediated by chronic liver injury (Figure 2). We induced *Cebpb* KO by treating *Cebpb-*floxed male mice with AAV-TBG.Cre or control AAV during the first week of 10 weeks of TAA exposure (Figure 2A). *Cebpb* KO did not alter the weight of the mice (Figure 2B). We found a mild elevation of prothrombin time PT and international normalized ratio in KO mice but no difference in ALT levels (Figure 2C, D). We found that *Cebpb* KO in TAA-treated mice resulted in increased fibrosis development (Figure 2E) and profibrotic and proinflammatory gene expression (Figure 2F). Taken together, these data suggest that C/EBPβ protects from liver fibrosis in the TAA model.

### C/EBPβ promotes weight gain and liver steatosis in high-fat diet–fed male mice

**FIGURE 3:**
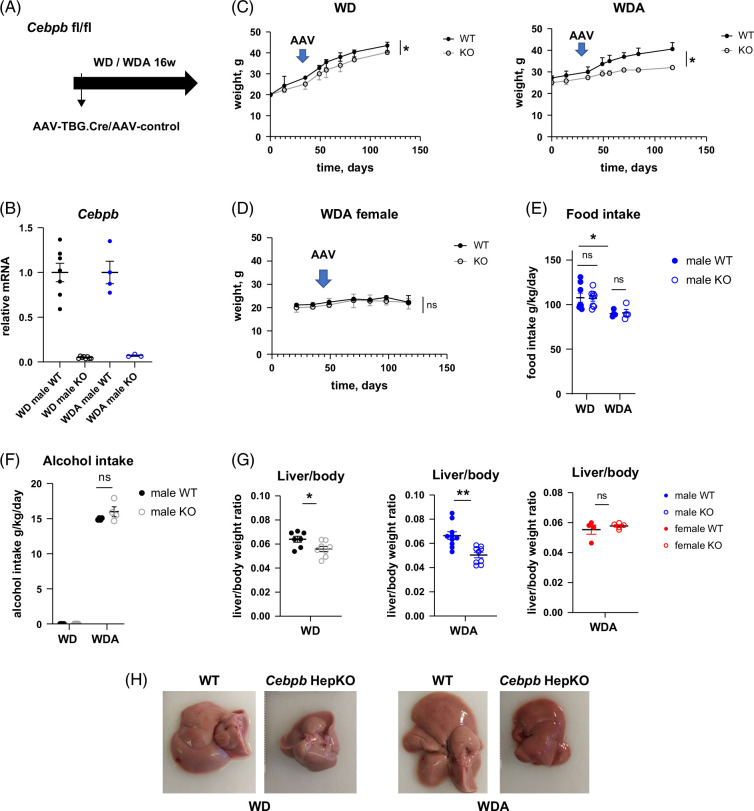
Hepatocyte-specific *Cebpb* knockout controls weight gain and liver size in male mice fed high-fat diet and alcohol. (A) Seven- to 8-week-old *Cebpb-*floxed mice were fed ad libitum western diet with or without alcohol in the drinking water for 16 weeks as indicated. Two weeks after the start of feeding, mice received 10^11^ gc/mouse of AAV-TBG-Cre or AAV-TBG-control. (B) Relative gene expression in WT and KO male mice. N ≥3 per group. (C, D) Weight changes over time in male mice fed WD or WDA (C), and female mice fed WDA (D). N ≥3 per group. *, *p*<0.05. (E, F) Food intake and alcohol intake in male mice 1 week after AAV injection. N ≥3 per group. *, *p*<0.05. (G) Liver-to-body weight ratio in male and female mice at the end of the experiment N ≥3 per group. *, *p*<0.05; **, *p*<0.01. (H) Representative images of livers of male mice at the end of the feeding. Abbreviations: AAV, adeno-associated virus; KO, knockout; WD, western diet; WDA, western diet with alcohol; WT, wild type.

To evaluate the role of C/EBPβ in high-fat diet and alcohol-induced liver disease, we fed male *Cebpb-*floxed mice WD or WD with 20% alcohol in the drinking water for 16 weeks. Shortly after the initiation of the diet, mice received AAV-TBG-Cre or control AAV (Figure 3A). At the end of the feeding, we confirmed that AAV-Cre nearly eliminated *Cebpb* mRNA expression (Figure 3B). *Cebpb* KO resulted in decreased weight gain in both groups of male mice (Figure 3C). We next tested whether we similar phenotype was present in female mice fed WD and alcohol (Figure 3D). In contrast to male mice, females fed WDA did not show differences in weight gain after *Cebpb* KO (Figure 3D). We analyzed whether food intake was altered in KO male mice and found that alcohol in the drinking water reduced high-fat diet intake as we previously reported^18^; however, there was no difference in food intake between genotypes (Figure 3E). Similarly, WT and KO mice had equal alcohol intake (Figure 3F), suggesting that the disease development in these mice is not associated with altered food/water intake. Differences in weight gain correlated with the change in liver/body weight ratios at the end of the feeding. While male *Cebpb* KO mice fed WD or WDA had reduced liver size and liver/body weight ratios compared to WT controls (Figure 3G, H), we found no difference in female mice fed WDA (Figure 3G).

**FIGURE 4:**
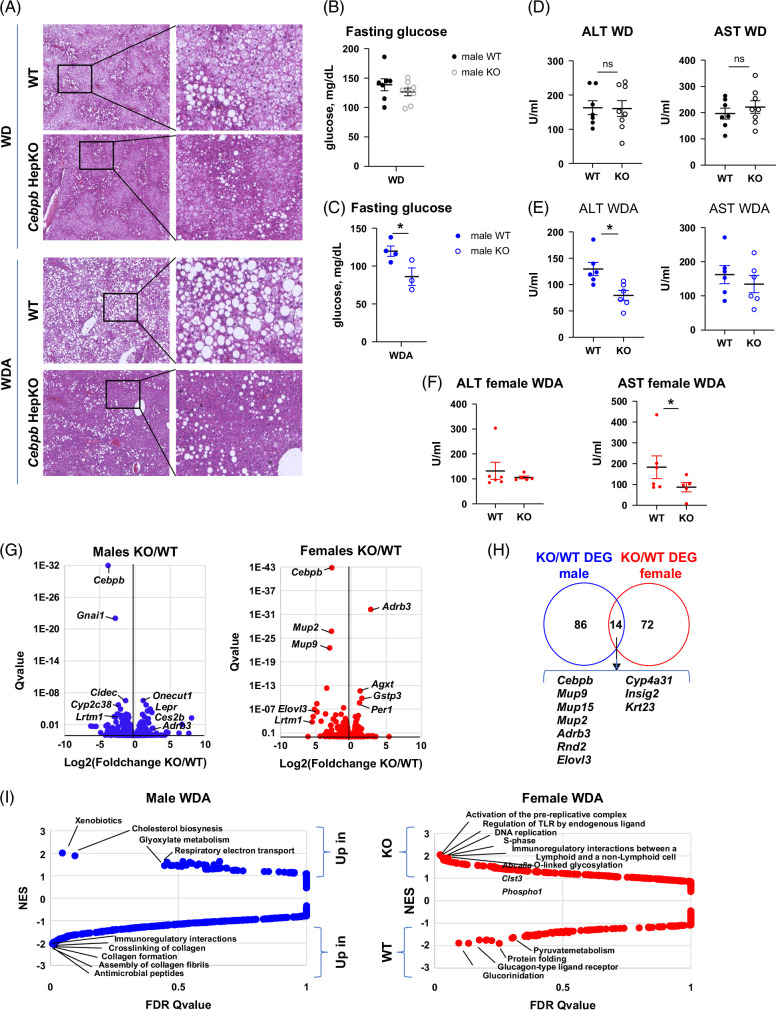
Hepatocyte-specific *Cebpb* knockout protects from liver steatosis and liver injury in male mice fed high-fat diet and alcohol. Seven- to 8-week-old *Cebpb-*floxed mice were fed ad libitum western diet with or without alcohol in the drinking water for 16 weeks as indicated. Two weeks after the start of feeding, mice received 10^11^ gc/mouse of AAV-TBG-Cre or AAV-TBG-control. (A) Representative images of H&E staining of livers of male mice at the end of the feeding. (B, C) Fasting glucose levels at the end of the experiment in male mice. N ≥3 per group. *, *p*<0.05. (D–F) Serum ALT and AST levels in male mice fed WD (D), male mice fed WDA (E) and female mice fed WDA (F) at the end of the experiment N ≥3 per group. *, *p*<0.05. (G) Whole liver RNA was isolated from male and female mice fed WDA and subjected to RNA sequencing. Volcano plot of differentially regulated genes in wild-type and knockout male and female mice fed WDA diet. N=3 mice per group. (H) Common differentially expressed genes regulated by C/EBPβ in males and females. (I) Pathway enrichment in differentially regulated genes using Gene Set Enrichment analysis regulated by C/EBPβ in males and females as indicated. Abbreviations: KO, knockout; NES, normalized enrichment score; WD, western diet; WDA, western diet with alcohol; WT, wild type.

Next, we used hematoxylin and eosin staining to evaluate liver steatosis. WDA-fed male mice showed increased liver steatosis, as previously reported.^18^ *Cebpb* KO male mice showed dramatically reduced liver steatosis in both diet groups (Figure 4A). Next, we measured fasting glucose (Figure 4B, C) and ALT/AST levels in male mice at the end of the experiment (Figure 4D, E). *Cebpb* KO reduced several of the adverse effects of the WDA diet with significantly lower fasting blood glucose levels and lower serum ALT, but the KO did not produce this protection in WD diet-fed mice. These results suggest that the beneficial effects of the *Cebpb* KO were at least partially specific to the presence of alcohol in the diet. We found that in female mice fed the WDA diet *Cebpb* KO reduced liver injury marker—AST levels (Figure 4F), suggesting that it may have some protective effect on ALD development in females as well.

To further understand the changes induced by *Cebpb* KO in male and female mice, we performed bulk RNA-seq analysis of WT and *Cebpb* KO alcohol-fed male and female mouse livers (Figure 4G). We found that very few genes were common between the differentially regulated genes in males and females (Figure 4H). Among these genes were *Cebpb* itself and adrenergic receptor *Adrb3*, an upstream regulator of C/EBPβ that is modulated by C/EBPβ transcriptional activity,^23^ as well as genes such as *Elovl3* and *Insig2*, which are involved in liver metabolism. Further analysis of pathways perturbed by *Cebpb* KO in the liver showed that in male mice, *Cebpb* KO promotes mitochondrial gene expression, which could be associated with reduced weight gain, and suppresses genes involved in collagen synthesis, crosslinking, and assembly (Figure 4I). In contrast, in female mice, *Cebpb* KO promoted replication and immune-related pathways and had no effect on fibrosis-related pathways (Figure 4I).

### C/EBPβ promotes alcohol-induced liver fibrosis in males

**FIGURE 5:**
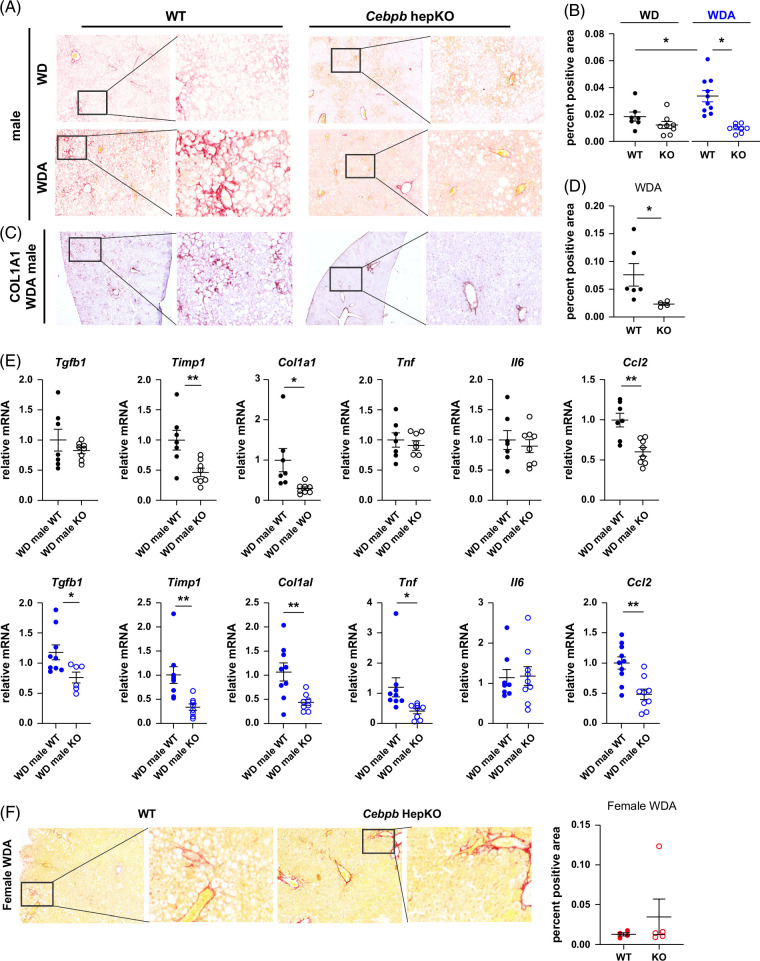
Hepatocyte-specific *Cebpb* knockout prevents liver fibrosis development in male mice. Seven- to 8-week-old *Cebpb-*floxed male and female mice were fed ad libitum western diet with or without alcohol in the drinking water for 16 weeks. Two weeks after the start of feeding mice received 10^11^ gc/mouse of AAV-TBG-Cre or AAV-TBG-control. (A) Representative images of Sirius Red staining in male mice fed WD or WDA. (B) Sirius red positive area in male mice. N ≥3 per group. *, *p*<0.05; **, *p*<0.01. (C) Representative images of COL1A1 staining in male mice fed WDA. (D) COL1A1-positive area in male mice fed WDA. N ≥3 per group. *, *p*<0.05. (E) Relative liver mRNA levels in male mice fed WD (black) or WDA (blue). N ≥3 per group. *, *p*<0.05; **, *p*<0.01. (F) Representative images of Sirius Red staining in female mice fed WDA. (Right) Sirius red positive area in female mice fed WDA. Abbreviations: KO, knockout; WD, western diet; WDA, western diet with alcohol; WT, wild type.

In agreement with these data, we found that *Cebpb* KO in male mice abolished alcohol-induced fibrosis development (Figure 5A, B). We found that in males 16 weeks of WD feeding did not produce detectable levels of fibrosis in both WT and KO mice. In WD and alcohol-fed WT mice, alcohol feeding resulted in a significant increase in liver fibrosis, which was abolished by *Cebpb* KO. We confirmed these findings using immunohistochemistry staining for Collagen 1A1 protein (Figure 5C, D). We found that in alcohol-fed mice, there is strong staining for COL1A1 in the pericellular areas throughout the liver parenchyma. There was no pericellular COL1A1 protein staining in *Cebpb* KO mice (Figure 5C, D). These data correlated with reduced *Tgfb1, Timp1*, and *Col1a1* mRNA in *Cebpb* KO male mice fed WDA (Figure 5E). In mice fed WD, only we similarly found a decrease of *Timp1* and *Col1a1* mRNA in *Cebpb* KO male mice. In addition, we found a decrease in *Tnf* and *Ccl2* mRNA in *Cebpb* KO mice, suggesting that reduced inflammation could contribute to the protection in KO mice. In contrast, in females, *Cebpb* KO had no effect on fibrosis (Figure 5F).

### C/EBPβ modulates hepatocyte macrophage cross talk

**FIGURE 6:**
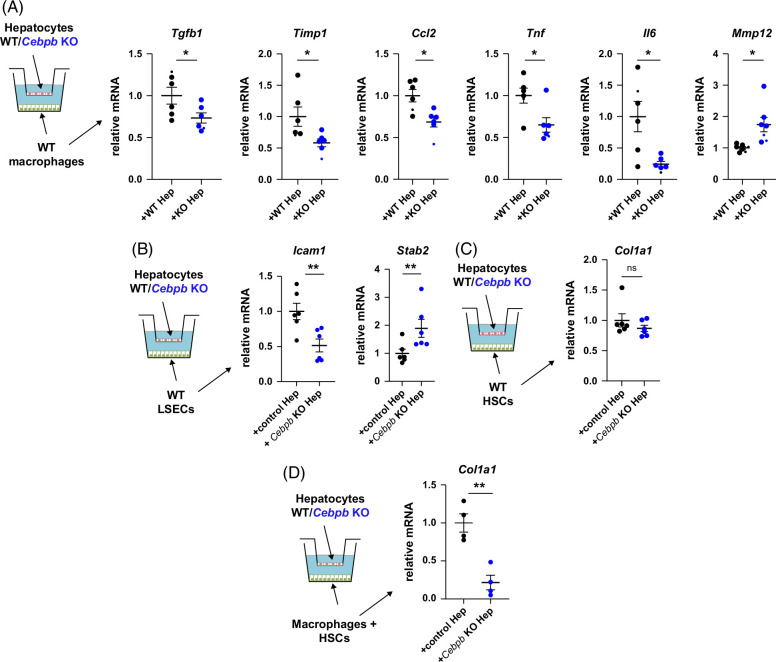
C/EBPβ inhibition promotes proresolving phenotype in liver macrophages. Wild-type (WT) or *Cebpb* knockout (KO) hepatocytes isolated from male mice were co-cultured with wild-type liver macrophages (A), LSEC (B), HSC (C), or macrophages and HSC together (D) in a trans-well co-culture system. Relative mRNA in nonparenchymal cells, N=4–6 per group. *, *p*<0.05; **, *p*<0.01. Abbreviations: KO, knockout; WD, western diet; WDA, western diet with alcohol; WT, wild type.

We next tested the effect of hepatocyte *Cebpb* KO on the phenotype of nonparenchymal cells (macrophages, endothelial cells, and HSCs) to examine cellular cross talk involved in improved fibrosis resolution. We isolated hepatocytes and nonparenchymal cells from male mice since we observed the protection in male mice only, and induced *Cebpb* KO by Cre recombinase expression. We found that the greatest effect of hepatocyte *Cebpb* KO was on liver macrophages (Figure 6A–C). Co-culture with *Cebpb* KO hepatocytes reduced proinflammatory and profibrotic gene expression in macrophages and increased expression of genes associated with the proresolving phenotype, such as *Mmp12*. In addition, co-culture with *Cebpb* KO hepatocytes reduced the adhesion molecule *Icam1* in LSECs (Figure 6B) but had no effect on HSCs (Figure 6C). To test whether macrophages could mediate hepatocyte effect on stellate cell activation, we combined these 2 cell types together. We found that co-culture with *Cebpb* KO hepatocytes reduced HSC activation when macrophages were present (Figure 6D).

Taken together, these data suggest that C/EBPβ activation in hepatocytes promotes profibrotic and proinflammatory changes in liver macrophages, thus modulating fibrosis development.

### C/EBPβ modulates hepatocyte macrophage cross talk via HDL remodeling

**FIGURE 7:**
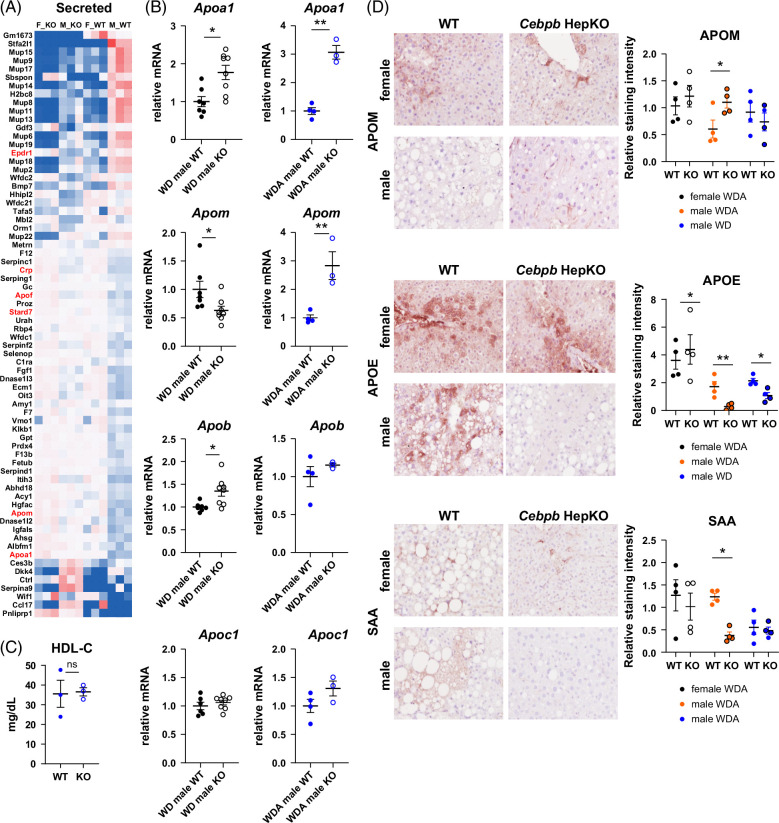
C/EBPβ promotes HDL-associated protein dysregulation. (A) Differentially regulated genes encoding secreted proteins in wild-type and knockout male and female mice fed WDA diet. N=3 mice per group. Lipid binding associated genes are in red. (B) Relative mRNA of apolipoprotein genes in male mice fed WD (black) or WDA (blue). N=3–8 per group. *, *p*<0.05; **, *p*<0.01. (C) HDL-C levels in serum of WDA-fed male mice. (D) Representative images of APOM, APOE, and SAA staining in male and female mice fed WDA. (Right) Relative staining intensity in female mice fed WDA (black), male mice fed WDA (orange), or WD (blue). N=4 per group. *, *p*<0.05; **, *p*<0.01. Abbreviations: APOE, Apolipoprotein E; APOM, Apolipoprotein M; KO, knockout; NES, normalized enrichment score; SAA, serum amyloid A; WD, western diet; WDA, western diet with alcohol; WT, wild type.

To assess possible mediators of cell-cell communication between hepatocytes and macrophages, we analyzed secreted proteins that showed differential gene expression in KO males (Figure 7A). We found that genes encoding several components of HDL, such as *Apoa1* and *Apom*, were increased in KO males while other lipid-associated genes were downregulated. Interestingly, in female mice, *Apoa1* and *Apom* levels were upregulated compared to male mice and were not altered by *Cebpb* KO (Figure 7A). We assessed several apolipoprotein levels in WT and KO male mice fed WD or WDA (Figure 7B). We found that *Apoa1* and *Apom* levels were the most upregulated in male KO mice fed WDA. Interestingly, this upregulation did not affect serum HDL-C levels in KO mice (Figure 7C). We confirmed that APOM protein levels were significantly upregulated in male KO mice, in contrast, in female mice APOM levels were elevated compared to male mice and did not further increase in KO mice (Figure 7D). Next, we assessed the HDL protein components that are associated with inflammatory HDL, SAA, and APOE. RNA-seq data suggested that *Saa1*, *Saa2*, and *Apoe* genes were slightly downregulated in *Cebpb* KO males; however, it did not reach the statistical significance cutoff. We next assessed their protein levels (Figure 7D). We found that in male mice, both SAA and APOE levels were reduced in the WDA group, and no significant difference was observed in females. Taken together, these data suggest that C/EBPβ regulates HDL protein levels that skew it toward a more proinflammatory phenotype.

**FIGURE 8:**
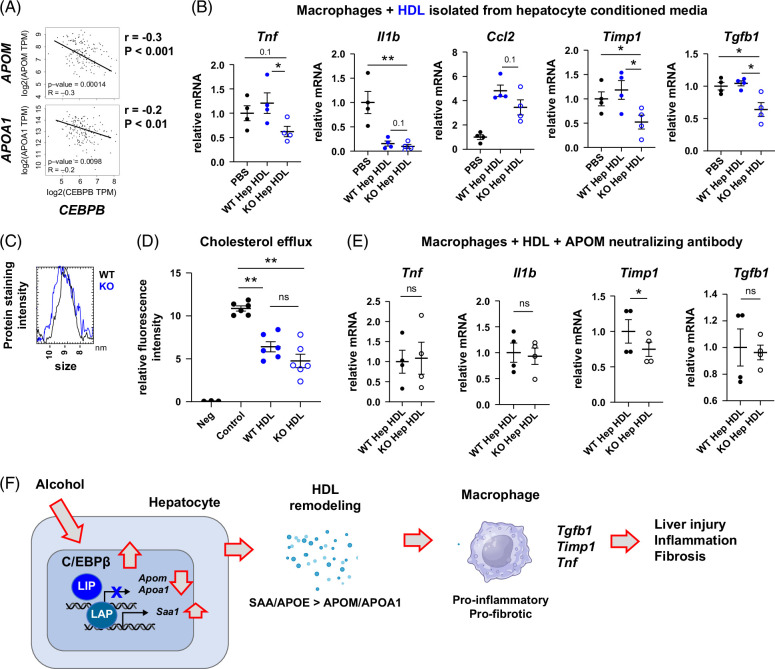
C/EBPβ inhibition promotes proresolving phenotype in liver macrophages via HDL remodeling. (A) Correlation between *CEBPB* and *APOM* or *APOA1* in human liver samples. (B) Wild-type (WT) or *Cebpb* knockout (KO) hepatocytes (isolated from male mice) conditioned media HDL was used to treat macrophages at a final concentration of 20 µg/mL (protein). HDL yield from WT and KO hepatocytes was similar between genotypes. Relative mRNA in macrophages after 24 hours, N=4–6 per group. *, *p*<0.05; **, *p*<0.01. (C) Non-denaturing gel electrophoresis analysis in 0.9% agarose followed by Coomassie staining of HDL isolated from WT and KO mice. Protein staining intensity profile of HDL peaks for WT (black) and KO (blue) HDL. (D) HDL at 20 µg/mL was used to treat wild-type peritoneal macrophages preloaded with BODIPY-cholesterol. Fluorescence intensity of intracellular BODIPY-cholesterol after 24 hours of incubation with HDL. Neg—negative control without BODIPY-cholesterol. Control—no HDL added. (E) HDL was used to treat macrophages at a final concentration of 20 µg/mL (protein) in the presence of APOM neutralizing antibody (1 µg/mL). Relative mRNA in macrophages after 24 hours, N=4–6 per group. *, *p*<0.05 by paired *t* test. (F) Model of C/EBPβ-dependent fibrosis development. Abbreviations: APOM, Apolipoprotein M; C/EBPβ, CCAAT enhancer binding protein beta; KO, knockout; LAP, liver-enriched activating protein; LIP, liver-enriched inhibitory protein; SAA, serum amyloid A; WT, wild type.

To test the relevance of this observation in humans, we assessed the correlation between *CEBPB* and *APOA1* and *APOM* using TCGA and GTEx databases (Figure 8A). We found that there was a significant negative correlation between *CEBPB* and *APOA1* or *APOM* in liver samples, suggesting that C/EBPβ regulates HDL function in humans as well.

Next, we tested whether HDL remodeling induced by C/EBPβ contributes to hepatocyte-macrophage cross talk. We isolated HDL from WT or *Cebpb* KO hepatocytes from male mice and used it to treat macrophages (Figure 8B). We found that KO hepatocyte-produced HDL can suppress macrophage *Tnf, Timp1*, and *Tgfb1* compared to WT hepatocyte HDL. We confirmed that these functional changes were not associated with HDL size change (Figure 8C) or its ability to induce cholesterol efflux in macrophages (Figure 8D). However, it depended on the presence of APOM. APOM neutralizing antibody abolished the ability of KO cells-produced HDL to suppress proinflammatory (*Tnf*) and profibrotic (*Tgfb1*) gene expression in macrophages (Figure 8E).

Taken together, these data suggest that HDL remodeling induced by C/EBPβ promotes proinflammatory and profibrotic signaling in macrophages, thus contributing to alcohol-induced inflammation and fibrosis development (Figure 8F).

## DISCUSSION

Environmental exposures such as alcohol and high-fat diet can promote long-lasting epigenetic changes that can affect disease progression. Early epigenetic changes induced in response to environmental changes are likely involved in modulating the expression of genes important for adaptation to environmental exposures.^4^,^24^,^25^ However, growing evidence suggests that long-term accumulation of epigenetic changes due to chronic exposures results in harmful gene expression changes that can promote disease progression.^26^,^27^,^28^,^29^ In the case of ALD, alcohol-induced epigenetic changes promote liver inflammation,^26^,^27^,^28^ loss of hepatocyte differentiated function,^25^,^30^,^31^ liver fibrosis^32^ and eventually HCC.^29^,^30^,^33^

In this work, we took an unbiased approach to define the TF drivers of alcohol-induced changes in liver cells using our previously published single-cell assay for transposase-accessible chromatin with sequencing data set. Specifically in hepatocytes, we identified that epigenetic changes were associated with activation of the C/EBPβ TF. The role of liver C/EBPβ in metabolic-associated or ALD has not been previously recognized. C/EBPβ has a known function in liver regeneration after partial hepatectomy and recently we identified C/EBPβ as a key driver in acute-on-chronic liver failure, where it antagonizes HNF4α mediated transcription and drives the suppression of hepatocyte metabolic and synthetic function.^12^ In addition, C/EBPβ is known to regulate inflammatory responses, acute phase response gene expression, growth hormone response, and glucose signaling. These data agree with our finding of altered glucose/insulin signaling in the livers of *Cebpb* KO mice.

In addition, we found that C/EBPβ is essential for alcohol and high-fat diet-induced liver fibrosis development, liver inflammation, and steatosis. Hepatocyte specific *Cebpb* KO mice were protected from fibrosis development, had less liver steatosis, and less liver injury and inflammation. To define the potential mechanism of C/EBPβ-mediated regulation of fibrosis development and resolution, we examined cell-cell communication between WT or *Cebpb* KO hepatocytes and nonparenchymal cells. We found that *Cebpb* KO reduced profibrotic and proinflammatory and increased proresolving gene expression in liver macrophages, suggesting that altered macrophage phenotype could be the driver of phenotypic change in *Cebpb* KO mice.

We found that *Cebpb* KO during ALD development was protective in male mice but not in female mice. This was associated with a mild change in weight gain in KO males on a high-fat WD. This effect was further exacerbated by alcohol feeding. Alcohol increased fibrosis development and inflammation in WT males, however KO male mice remained protected and showed no alcohol-dependent effects. In contrast, female KO mice fed WD and alcohol showed no difference in weight gain and fibrosis, and a small decrease in liver injury marker AST, suggesting that in WDA model, ALD development in females is induced by other mechanisms in agreement with our previous studies.^18^,^34^,^35^ Future studies are necessary to define sex-divergent roles of C/EBPβ in the liver and whether C/EBPβ inhibition is equally beneficial for male and female patients with ALD.

To further define the mechanism of cell-cell communication changes induced by C/EBPβ KO in hepatocytes, we explored secreted factors in gene expression changes in KO mice. We found that several factors associated with HLD were altered in KO mice, including APOA1 and APOM. APOA1 is the critical protein constituent of HDL and is required for HDL functions such as removing excess cholesterol from peripheral tissues and its anti-inflammatory properties.^36^ APOM is an HDL-associated protein that accounts for ~5% of HDL protein content. Despite low abundance, it has significant metabolic effects.^37^ APOM enhances cholesterol efflux, as well as the anti-inflammatory functions of HDL. APOM was recently reported to be protective against tissue injury and fibrosis in the kidney, liver, and lung, endotoxin-induced inflammation, and tumor development,^38^,^39^,^40^,^41^,^42^ suggesting that APOM changes could contribute to KO mice phenotype. We found that *Apoa1* and *Apom* were specifically upregulated in WDA-fed male KO mice but not female KO mice.

In contrast to APOA1 and APOM, 2 other HDL components, SAA or serum amyloid A and APOE, were downregulated in KO mice. SAA is an acute phase response gene,^43^,^44^,^45^ which can displace APOA1 from HDL particles. SAA can reduce HDL’s ability to promote cholesterol efflux and increase the uptake of cholesteryl ester by the liver via SR-BI.^37^,^43^,^46^ It has both proinflammatory and anti-inflammatory properties. In metabolic-associated steatotic liver disease models, deficiency of *Saa1/Saa2* in mice due to genetic KO or shRNA knockdown ameliorates steatohepatitis, suggesting that SAA is pathogenic in high-fat diet–induced liver disease. SAA genes *Saa1, Saa2, Saa3*, and *Saa4* are well-known targets of C/EBPβ.^45^,^47^ We found that SAA levels were decreased in C/EBPβ KO mice which correlated with reduced inflammation, steatosis, and fibrosis in KO mice. Finally, we identified that C/EBPβ-induced HDL remodeling altered HDL properties in inducing proinflammatory and profibrotic changes in macrophages, thus contributing to pathogenic cell-cell cross talk in the liver after alcohol feeding.

Altered HDL composition likely contributes to sex differences observed in this model. Sex differences in HDL are well recognized. Women generally have higher HDL cholesterol and lower triglycerides, higher HDL particle concentration, and more APOA1, APOB, and altered APOE function. We found that in the presence of alcohol, C/EBPβ reduces APOA1 and APOM, specifically in male mice, while in females these 2 genes are C/EBPβ independent, which correlated with the protective effect of *Cebpb* KO in males but not in females. In addition, we found that APOM levels were slightly reduced while SAA levels were increased by alcohol feeding in male mice, which correlated with alcohol-induced fibrosis development, suggesting that C/EBPβ-SAA/APOM axis could contribute to alcohol-specific effects. We found that APOM depletion abolishes the protective effect of KO hepatocyte HDL, suggesting that APOM regulation is critical for HDL-mediated protection. Further studies are necessary to define the contribution of individual HDL components to ALD development.

C/EBPβ is encoded by an intron-free gene and has three isoforms that differ in translation start sites, including 2 activators (LAP* and LAP) and a repressor (LIP).^9^,^16^,^48^ We found that both isoforms were induced in WDA mice and in mice treated with TAA (Figure 1D). However, the role of LIP and LAP isoforms in ALD and HDL remodeling is not clear. It is likely that the LIP isoform is the main contributor of APOM/APOA1 suppression, and LAP is involved in SAA induction. Previous studies using the ChIP-sequencing approach reported C/EBPβ binding to both *Apoa1* and *Saa1* genes,^6^ suggesting that these are direct targets of C/EBPβ. However, the role of direct binding for HDL protein regulation in ALD needs further validation.

Our data suggest that C/EBPβ is a promising treatment target for ALD. Since C/EBPβ levels are low under normal conditions, C/EBPβ inhibition could be a promising therapeutic strategy to promote fibrosis resolution in patients with ALD. Several peptides have been shown to inhibit C/EBPβ TF activity, specifically Dpep, Bpep, and ST101. Among the actions reported for these peptides is stimulation of ubiquitin-dependent proteasomal degradation of C/EBPβ.^49^,^50^ Both Dpep and ST101 were extensively tested in vivo in animal models and showed positive results with low toxicity. ST101 is currently in a clinical trial for glioma. Our data suggest that peptide-mediated C/EBPβ inhibition could be a promising strategy for ALD treatment.

In summary, this work illustrates how alcohol-induced C/EBPβ upregulation promotes HDL protein changes in males that result in altered cell-cell communication in the liver, which in turn promotes inflammation and fibrosis. While many details of these interactions remain to be determined, they open a number of potential possible therapeutic interventions to promote the resolution of ALD.

## References

[1] Osna NA Donohue TM Jr Kharbanda KK. Alcoholic liver disease: Pathogenesis and current management. Alcohol Res. 2017;38:147–61. 28988570 10.35946/arcr.v38.2.01 PMC5513682

[2] Zhang R Zhong B He J Yang X He M Zeng W. Single-cell transcriptomes identifies characteristic features of mouse macrophages in liver Mallory-Denk bodies formation. Exp Mol Pathol. 2022;127:104811. 35850229 10.1016/j.yexmp.2022.104811

[3] Xiong X Kuang H Ansari S Liu T Gong J Wang S. Landscape of intercellular crosstalk in healthy and NASH liver revealed by single-cell secretome gene analysis. Mol Cell. 2019;75:644–660.e5. 31398325 10.1016/j.molcel.2019.07.028 PMC7262680

[4] Zhao J Adams A Weinman SA Tikhanovich I. Hepatocyte PRMT1 protects from alcohol induced liver injury by modulating oxidative stress responses. Sci Rep. 2019;9:9111. 31235809 10.1038/s41598-019-45585-2 PMC6591482

[5] Das S Ge X Han H Desert R Song Z Athavale D. The integrated “multiomics” landscape at peak injury and resolution from alcohol-associated liver disease. Hepatol Commun. 2022;6:133–60. 34558855 10.1002/hep4.1793 PMC8710802

[6] Jakobsen JS Waage J Rapin N Bisgaard HC Larsen FS Porse BT. Temporal mapping of CEBPA and CEBPB binding during liver regeneration reveals dynamic occupancy and specific regulatory codes for homeostatic and cell cycle gene batteries. Genome Res. 2013;23:592–603. 23403033 10.1101/gr.146399.112 PMC3613577

[7] Wang H Peiris TH Mowery A Le Lay J Gao Y Greenbaum LE. CCAAT/enhancer binding protein-beta is a transcriptional regulator of peroxisome-proliferator-activated receptor-gamma coactivator-1alpha in the regenerating liver. Mol Endocrinol. 2008;22:1596–1605. 18467525 10.1210/me.2007-0388 PMC2453599

[8] Wang B Gao C Ponder KP. C/EBPbeta contributes to hepatocyte growth factor-induced replication of rodent hepatocytes. J Hepatol. 2005;43:294–302. 15922473 10.1016/j.jhep.2005.02.029

[9] Luedde T Duderstadt M Streetz KL Tacke F Kubicka S Manns MP. C/EBP beta isoforms LIP and LAP modulate progression of the cell cycle in the regenerating mouse liver. Hepatology. 2004;40:356–365. 15368440 10.1002/hep.20333

[10] He L Ronis MJ Badger TM. Ethanol induction of class I alcohol dehydrogenase expression in the rat occurs through alterations in CCAAT/enhancer binding proteins beta and gamma. J Biol Chem. 2002;277:43572–43577. 12213809 10.1074/jbc.M204535200

[11] Trautwein C Rakemann T Pietrangelo A Plumpe J Montosi G Manns MP. C/EBP-beta/LAP controls down-regulation of albumin gene transcription during liver regeneration. J Biol Chem. 1996;271:22262–22270. 8703043 10.1074/jbc.271.36.22262

[12] Elias G Schonfeld M Saleh S Parrish M Barmanova M Weinman SA. Sepsis-induced endothelial dysfunction drives acute-on-chronic liver failure through Angiopoietin-2-HGF-C/EBPbeta pathway. Hepatology. 2023;78:803–19. 36943063 10.1097/HEP.0000000000000354 PMC10440279

[13] Gao Y Sun W Shang W Li Y Zhang D Wang T. Lnc-C/EBPbeta negatively regulates the suppressive function of myeloid-derived suppressor cells. Cancer Immunol Res. 2018;6:1352–63. 30171135 10.1158/2326-6066.CIR-18-0108

[14] Simpson-Abelson MR Hernandez-Mir G Childs EE Cruz JA Poholek AC Chattopadhyay A. CCAAT/Enhancer-binding protein beta promotes pathogenesis of EAE. Cytokine. 2017;92:24–32. 28088614 10.1016/j.cyto.2017.01.005 PMC5337143

[15] Maekawa T Hosur K Abe T Kantarci A Ziogas A Wang B. Antagonistic effects of IL-17 and D-resolvins on endothelial Del-1 expression through a GSK-3beta-C/EBPbeta pathway. Nat Commun. 2015;6:8272. 26374165 10.1038/ncomms9272 PMC4573473

[16] Cui TX Lin G LaPensee CR Calinescu AA Rathore M Streeter C. C/EBPbeta mediates growth hormone-regulated expression of multiple target genes. Mol Endocrinol. 2011;25:681–693. 21292824 10.1210/me.2010-0232 PMC3063086

[17] Zhu S Yoon K Sterneck E Johnson PF Smart RC. CCAAT/enhancer binding protein-beta is a mediator of keratinocyte survival and skin tumorigenesis involving oncogenic Ras signaling. Proc Natl Acad Sci USA. 2002;99:207–212. 11756662 10.1073/pnas.012437299 PMC117540

[18] Schonfeld M O’Neil M Villar MT Artigues A Averilla J Gunewardena S. A Western diet with alcohol in drinking water recapitulates features of alcohol-associated liver disease in mice. Alcohol Clin Exp Res. 2021;45:1980–93. 34523155 10.1111/acer.14700 PMC9006178

[19] Zhao E Ilyas G Cingolani F Choi JH Ravenelle F Tanaka KE. PRMT1 and JMJD6 dependent arginine methylation regulate HNF4alpha expression and hepatocyte proliferation. Hepatology. 2017;66:922–935. 28470665 10.1002/hep.29244 PMC5570662

[20] Schonfeld M O’Neil M Weinman SA Tikhanovich I. Alcohol-induced epigenetic changes prevent fibrosis resolution after alcohol cessation in mice. Hepatology. 2024;80:119–35. 37943941 10.1097/HEP.0000000000000675 PMC11078890

[21] Stuart T Srivastava A Madad S Lareau CA Satija R. Single-cell chromatin state analysis with Signac. Nat Methods. 2021;18:1333–41. 34725479 10.1038/s41592-021-01282-5 PMC9255697

[22] Ghosh Dastidar S Warner JB Warner DR McClain CJ Kirpich IA. Rodent models of alcoholic liver disease: Role of binge ethanol administration. Biomolecules. 2018;8:3. 29342874 10.3390/biom8010003 PMC5871972

[23] Zhou Z Shu Y Bao H Han S Liu Z Zhao N. Stress-induced epinephrine promotes epithelial-to-mesenchymal transition and stemness of CRC through the CEBPB/TRIM2/P53 axis. J Transl Med. 2022;20:262. 35672760 10.1186/s12967-022-03467-8 PMC9172202

[24] Nagy LE. The role of innate immunity in alcoholic liver disease. Alcohol Res. 2015;37:237–250. 26695748 10.35946/arcr.v37.2.08 PMC4590620

[25] Qin Y Grimm SA Roberts JD Chrysovergis K Wade PA. Alterations in promoter interaction landscape and transcriptional network underlying metabolic adaptation to diet. Nat Commun. 2020;11:962. 32075973 10.1038/s41467-020-14796-x PMC7031266

[26] Vidali M Stewart SF Rolla R Daly AK Chen Y Mottaran E. Genetic and epigenetic factors in autoimmune reactions toward cytochrome P4502E1 in alcoholic liver disease. Hepatology. 2003;37:410–419. 12540792 10.1053/jhep.2003.50049

[27] Mandrekar P. Epigenetic regulation in alcoholic liver disease. World J Gastroenterol. 2011;17:2456–2464. 21633650 10.3748/wjg.v17.i20.2456 PMC3103803

[28] Curtis BJ Zahs A Kovacs EJ. Epigenetic targets for reversing immune defects caused by alcohol exposure. Alcohol Res. 2013;35:97–113. 24313169 10.35946/arcr.v35.1.11 PMC3860427

[29] French SW. Epigenetic events in liver cancer resulting from alcoholic liver disease. Alcohol Res. 2013;35:57–67. 24313165 10.35946/arcr.v35.1.07 PMC3860418

[30] Zhao J Adams A Roberts B O’Neil M Vittal A Schmitt T. Protein arginine methyl transferase 1- and Jumonji C domain-containing protein 6-dependent arginine methylation regulate hepatocyte nuclear factor 4 alpha expression and hepatocyte proliferation in mice. Hepatology. 2018;67:1109–26. 29023917 10.1002/hep.29587 PMC5826837

[31] Schonfeld M Averilla J Gunewardena S Weinman SA Tikhanovich I. Male-specific activation of lysine demethylases 5B and 5C mediates alcohol-induced liver injury and hepatocyte dedifferentiation. Hepatol Commun. 2022;6:1373–91. 35084807 10.1002/hep4.1895 PMC9134811

[32] Perugorria MJ Wilson CL Zeybel M Walsh M Amin S Robinson S. Histone methyltransferase ASH1 orchestrates fibrogenic gene transcription during myofibroblast transdifferentiation. Hepatology. 2012;56:1129–1139. 22488473 10.1002/hep.25754 PMC3430805

[33] Pocha C Xie C. Hepatocellular carcinoma in alcoholic and non-alcoholic fatty liver disease-one of a kind or two different enemies? Transl Gastroenterol Hepatol. 2019;4:72. 31728429 10.21037/tgh.2019.09.01 PMC6851438

[34] Schonfeld M Averilla J Gunewardena S Weinman SA Tikhanovich I. Alcohol-associated fibrosis in females is mediated by female-specific activation of lysine demethylases KDM5B and KDM5C. Hepatol Commun. 2022;6:2042–57. 35468265 10.1002/hep4.1967 PMC9315128

[35] Nataraj K Schonfeld M Rodriguez A Tikhanovich I. Protective role of 17beta-estradiol in alcohol-associated liver fibrosis is mediated by suppression of integrin signaling. Hepatol Commun. 2024;8:e0428. 38704651 10.1097/HC9.0000000000000428 PMC11073774

[36] van der Westhuyzen DR de Beer FC Webb NR. HDL cholesterol transport during inflammation. Curr Opin Lipidol. 2007;18:147–151. 17353662 10.1097/MOL.0b013e328051b4fe

[37] Kisilevsky R Manley PN. Acute-phase serum amyloid A: Perspectives on its physiological and pathological roles. Amyloid. 2012;19:5–14. 10.3109/13506129.2011.654294 22320226

[38] Ding BS Yang D Swendeman SL Christoffersen C Nielsen LB Friedman SL. Aging suppresses sphingosine-1-phosphate chaperone ApoM in circulation resulting in maladaptive organ repair. Dev Cell. 2020;53:677–90 e4. 32544390 10.1016/j.devcel.2020.05.024 PMC7607448

[39] Mousa H Thanassoulas A Zughaier SM. ApoM binds endotoxin contributing to neutralization and clearance by high density lipoprotein. Biochem Biophys Rep. 2023;34:101445. 36915826 10.1016/j.bbrep.2023.101445 PMC10006442

[40] Hajny S Christoffersen C. A novel perspective on the ApoM-S1P axis, highlighting the metabolism of ApoM and its role in liver fibrosis and neuroinflammation. Int J Mol Sci. 2017;18:1636. 28749426 10.3390/ijms18081636 PMC5578026

[41] Christoffersen C Benn M Christensen PM Gordts P Roebroek AJM Frikke-Schmidt R. The plasma concentration of HDL-associated apoM is influenced by LDL receptor-mediated clearance of apoB-containing particles. J Lipid Res. 2012;53:2198–204. 22826357 10.1194/jlr.P023697 PMC3435552

[42] Cheng G Zheng L. Regulation of the apolipoprotein M signaling pathway: A review. J Recept Signal Transduct Res. 2022;42:285–92. 34006168 10.1080/10799893.2021.1924203

[43] Hayat S Raynes JG. Acute phase serum amyloid A protein increases high density lipoprotein binding to human peripheral blood mononuclear cells and an endothelial cell line. Scand J Immunol. 2000;51:141–146. 10652160 10.1046/j.1365-3083.2000.00661.x

[44] Mullan RH Bresnihan B Golden-Mason L Markham T O’Hara R FitzGerald O. Acute-phase serum amyloid A stimulation of angiogenesis, leukocyte recruitment, and matrix degradation in rheumatoid arthritis through an NF-kappaB-dependent signal transduction pathway. Arthritis Rheum. 2006;54:105–114. 16385502 10.1002/art.21518

[45] Li X Liao WS. Cooperative effects of C/EBP-like and NF kappa B-like binding sites on rat serum amyloid A1 gene expression in liver cells. Nucleic Acids Res. 1992;20:4765–4772. 1408789 10.1093/nar/20.18.4765 PMC334230

[46] Vaisar T Tang C Babenko I Hutchins P Wimberger J Suffredini AF. Inflammatory remodeling of the HDL proteome impairs cholesterol efflux capacity. J Lipid Res. 2015;56:1519–1530. 25995210 10.1194/jlr.M059089 PMC4513993

[47] Li XX Liao WS. Expression of rat serum amyloid A1 gene involves both C/EBP-like and NF kappa B-like transcription factors. J Biol Chem. 1991;266:15192–15201. 1869549

[48] Liu Q Boudot A Ni J Hennessey T Beauparlant SL Rajabi HN. Cyclin D1 and C/EBPbeta LAP1 operate in a common pathway to promote mammary epithelial cell differentiation. Mol Cell Biol. 2014;34:3168–3179. 24912680 10.1128/MCB.00039-14 PMC4135606

[49] Zhou Q Greene LA. Dpep inhibits cancer cell growth and survival via shared and context-dependent transcriptome perturbations. Cancers (Basel). 2023;15:5318. 38001578 10.3390/cancers15225318 PMC10669862

[50] Zhou Q Sun X Pasquier N Jefferson P Nguyen TTT Siegelin MD. Cell-penetrating CEBPB and CEBPD leucine zipper decoys as broadly acting anti-cancer agents. Cancers (Basel). 2021;13:2504. 34065488 10.3390/cancers13102504 PMC8161188

